# S08-4 In terms of individual fitness, people with low cardiorespiratory fitness are physically the most active

**DOI:** 10.1093/eurpub/ckac093.043

**Published:** 2022-08-29

**Authors:** Pauliina Husu

**Affiliations:** UKK Institute for Health Promotion Research, Tampere, Finland

**Keywords:** physical fitness, accelerometer, Europe

## Abstract

**Background:**

Depending on cardiorespiratory fitness (CRF), people may perceive differently the exertion of incident physical activity (PA). It has been proposed to use thresholds relative to individual fitness in PA monitoring when it is feasible.

**Methods:**

1952 adults (803 men, 1149 women), aged 20-69 years participated in the FinFit2017 (EUPASMOS) -study. Their VO2max was predicted with 6 min walking test and they had accelerometer wear time at least four days with minimum of 24 h/day during seven consecutive days of measurement period. The participants were divided into CRF thirds by age groups and sex. Acceleration data was analyzed in 6s epochs and intensity in MET (metabolic equivalent) values was calculated for each epoch. MET values were smoothed with 1min exponential moving average. The epoch activity was classified into moderate-to-vigorous PA (MVPA) using both absolute (3.0MET) and individual (40% of the oxygen uptake reserve) thresholds. The accumulated MVPA time was calculated for bouts at least 0.1, 1.5, 3.0, 5.0, 10.0, 15.0, 20.0, 30.0 and 60.0 minutes. Population-weighted average of accumulated PA times in CRF-thirds and partial Spearman correlations controlled by age and sex between bout lengths and VO2max were calculated for both absolute and relative thresholds.

**Results:**

Participants in the lowest CRF-third accumulated the most (p > 0.05) MVPA time with relative threshold (22min (low); 15min (middle); 12min (high)) and the highest third (40min (low); 50min (middle); 56min (high)) with absolute threshold. The correlations were negative with individual thresholds, if short bouts were counted into accumulated time, and significant positive correlation (r = 0.048) was achieved only with at least 60.0min bouts. The correlations were significantly positive for all bout lengths with absolute thresholds and the highest correlation was achieved with at least 0.1min bouts (r = 0.295).

**Conclusions:**

The lowest fitness third was the most active when the PA intensity was analyzed using relative thresholds. Their PA was mostly accumulated from short, few minute bouts. Thus, they had to utilize more of their aerobic capacity on daily basis just to keep up with their daily routines and the peers.

